# Added value of contrast-enhanced mammography (CEM) in staging of malignant breast lesions—a feasibility study

**DOI:** 10.1186/s12957-020-01865-0

**Published:** 2020-05-21

**Authors:** Kristina Åhsberg, Anna Gardfjell, Emma Nimeus, Rogvi Rasmussen, Catharina Behmer, Sophia Zackrisson, Lisa Ryden

**Affiliations:** 1 Department of Surgery, Halland Hospital, 301 85 Halmstad, Sweden; 2 Institution of Clinical Sciences, Department of Surgery, Lund University, Lund, Sweden; 3 Department of Surgery, Blekinge Hospital, Karlskrona, Sweden; 4 Department of Surgery, Skåne University Hospital, Lund, Sweden; 5 Institution of Clinical Sciences, Department of Oncology, Lund University, Lund, Sweden; 6 Unilabs Breast Centre, Skåne University Hospital, Lund, Sweden; 7 Unilabs Breast Centre, Helsingborg Hospital, Helsingborg, Sweden; 8 Department of Imaging and Functional Medicine, Skåne University Hospital, Malmö, Sweden; 9 Diagnostic Radiology, Department of Translational Medicine, Lund University, Lund, Sweden

**Keywords:** Breast cancer, Preoperative staging, Contrast-enhanced mammography, CEM, Contrast-enhanced spectral mammography, CESM

## Abstract

**Objectives:**

The aim of this feasibility study was to evaluate the added value of contrast-enhanced mammography (CEM) in preoperative staging of malignant breast lesions, beyond standard assessment with digital mammography and ultrasound, as a base for a future prospective randomized trial.

**Materials and methods:**

Forty-seven patients, with confirmed or strongly suspected malignant breast lesions after standard assessment (digital mammography (DM) and ultrasound (US)), scheduled for primary surgery, were invited to undergo CEM as an additional preoperative procedure. The primary endpoint was change in treatment due to CEM findings, defined as mastectomy instead of partial mastectomy or contrariwise, bilateral surgery instead of unilateral or neoadjuvant treatment instead of primary surgery. Accuracy in tumour extent estimation compared to histopathology was evaluated by Bland-Altman statistics. Number of extra biopsies and adverse events were recorded.

**Results:**

In 10/47 patients (21%), findings from CEM affected the primary treatment. Agreement with histopathology regarding extent estimation was better for CEM (mean difference − 1.36, SD ± 18.45) in comparison with DM (− 4.18, SD ± 26.20) and US (− 8.36, SD ± 24.30). Additional biopsies were taken from 19 lesions in 13 patients. Nine biopsies showed malignant outcome. No major adverse events occurred.

**Conclusion:**

The feasibility of preoperative additional CEM was found to be satisfactory without any serious negative effects. Results imply an added value of CEM in preoperative staging of breast cancer. Further evaluation in larger prospective randomized trials is needed.

**Trial registration:**

ClinicalTrials.gov, NCT03402529. Registered 18 January 2018—retrospectively registered

## Background

Digital mammography (DM) is the standard imaging modality for breast cancer diagnostics [1]. Lower sensitivity of DM is related to, for example, high breast density [2–4], low patient age [2, 5] and lobular type of cancer [4]. The use of ultrasound (US) in the clinical setting is particularly helpful in characterizing palpable and non-palpable masses, guiding biopsies of non-palpable lesions and staging of nodal status in the axilla [6]. In addition, US has been shown to better estimate tumour size in comparison with DM [7, 8], but is inferior to DM regarding detection of DCIS [5]. Both DM and US have been found to underestimate the size of the lesion in comparison with histopathological examination [8]. It is important to correctly assess the extent of the malignant tumour in the preoperative planning for optimal surgical resection of the tumour area. Overestimation can lead to unnecessary mastectomies, and underestimation to reoperations due to inadequate margins of the tumour bed.

In cases of equivocal findings in DM and US, additional imaging may be warranted for correct preoperative staging. Dynamic, contrast-enhanced magnetic resonance imaging (MRI) has so far been the modality with best sensitivity for detecting invasive cancer, and it is not affected by breast density [2, 9]. During the past 5 years, contrast-enhanced mammography (CEM) has been introduced as another complementary method at several breast centres in Europe and in the USA. Protocols for CEM have been described in detail previously [1, 10, 11]. In short, CEM uses low- and high-energy standard DM views after administration of iodinated contrast medium. From these images, CEM software creates a subtracted image that highlights contrast uptake. Due to neoangiogenesis, tumours have a larger uptake of contrast agent than other tissue, making the tumours more pronounced compared to surrounding tissue. For DCIS, which is not commonly associated with neoangiogenesis, the hypothesis is that leaky basal membranes allow leakage of contrast into the second (interstitial space) and third fluid space (mammary ducts) [12].

Initial studies of CEM have shown improved extent estimation compared to DM [11, 13–16], even in the presence of microcalcifications [17]. In one study, CEM showed an improved extent estimation for 30 patients with biopsy-proven lobular cancer [18]. In two retrospective studies, CEM was found to change diagnostic and/or treatment strategy in 41/195 patients (21%) [10] and 20/101 patients (20%) [19], respectively. However, only observational studies have yet been performed, with relatively small cohorts and a selected patient material [3, 10, 20]. Previously published studies of estimated preoperative extent by CEM compared with the histopathological extent have included 30 to 118 patients [11, 13–17, 20]. There is a need of larger prospective randomized trials with CEM to improve the level of evidence regarding the diagnostic value, effects on staging and choice of treatment in breast cancer.

The aim of this study was to evaluate the feasibility of CEM, including potential related adverse events, in the preoperative clinical setting, as a basis for a larger prospective randomized trial, for evaluation of the added value of CEM in preoperative staging beyond standard assessment with mammography and ultrasound.

## Method

### Study population

Forty-seven patients, who had histologically confirmed (*n* = 46) or at only imaging (probability of malignancy code 5 at DM and US) strongly suspected (*n* = 1) malignant breast lesions and who were scheduled for primary surgery, were included in this feasibility study. Solid lesions as well as malignant microcalcifications were considered eligible. The primary treatment plan was established at the multidisciplinary team conference (MDT) from DM and US images before the patient met the surgeon for information of the diagnosis and study inclusion. Study patients underwent CEM as an additional procedure before operation. The CEM findings were taken into consideration and discussed at a second MDT with a potential change in treatment plan. The CEM procedure was scheduled during the normal waiting period before surgery and did not prolong the patients’ time to treatment. All patients had a malignant diagnosis in postoperative histopathology. Another 28 patients were identified as potential study participants at MDT, but were not included or excluded for reasons listed in Table 1.

**Table 1:** Characteristics of not included or excluded patients screened for eligibility in the trial

Reason	Number
Shortage of time between diagnosis and operation	7
Patient declined participation	4
Patient not considered suitable due to comorbidity	2
Other reason	3
Fulfilment of exclusion criteria	
*Allergy to iodinated contrast agent*	*1*
*Treatment with metformin*	*2*
*Elevated serum creatinine*	*4*
*Inability to comprehend study information*	*3*
*Neoadjuvant treatment as primary plan*	*2*
Total	28

### Inclusion and exclusion criteria

Inclusion criteria were confirmed or strongly suspected malignant lesion in breast, for which primary surgery was planned and a signed informed consent. Exclusion criteria were planned neoadjuvant treatment, ongoing pregnancy, breast feeding, allergy to iodinated contrast agent, treatment with metformin, renal failure or elevated serum creatinine, age < 18 years or > 80 years, ongoing thyrotoxicosis (upon suspicion, an additional blood sample of thyroid stimulating hormone (TSH) was taken) and inability to comprehend oral and written information regarding the study.

### Data collection

Medical records and a questionnaire were used to collect patient data concerning factors that potentially can affect image diagnostics and to identify contraindications for CEM: weight, height, age, ongoing pregnancy or breast feeding, medications including progesterone/estrogen (oral contraceptives, hormone replacement therapy) or anti-hormonal treatment (aromatase-inhibitors, tamoxifen), kidney disease, use of the antidiabetic medication metformin and allergy to iodinated contrast agent.

Information on cancer subtype (ductal/lobular/other), uni-/multifocal lesions, surrounding DCIS, histological grade, status of estrogen receptor (ER) and progesterone receptor (PgR), Her2 amplification and Ki67 percentage (histological marker related to proliferation) was collected from the postoperative pathological report. Histological total extent was also retrieved.

### Imaging procedures

DM and US were performed before study inclusion according to clinical standard. Breast density was graded from A to D according to BI-RADS® 5th edition [21], from the DM images by the radiologist after inclusion. The CEM examinations were performed at Unilabs Breast Centre in Lund on a Senographe Pristina mammography system equipped with SenoBright™ HD for CEM (GE Healthcare). The contrast medium used was Omnipaque 300 mg/ml, 1.5 mg/kg bodyweight (maximum 125 mg). It was injected by a peripheral venous catheter in the arm, preferably on the side of the body not affected by cancer. Injection time was about 30 s. After 1.45 min from the start of injection, compression was started. Dual imaging with high- and low-energy images at each projection was performed after 2 min in the following order: cranio-caudal (CC) projection of the unaffected side, CC projection of the affected side, mediolateral-oblique (MLO) projection of the affected side, MLO projection of the unaffected side, mediolateral (ML) projection of the affected side and finally ML projection of the unaffected side. All imaging had to be completed within 5 min (total time from start of injection maximally 7 min). Patients were observed after the contrast media injection to discover potential allergic reactions.

All CEM images were read by the same radiologist (RR). At the second MDT, all CEM images were reviewed once more. The reading of CEM was not blinded since the study was performed as a part of the clinical routine. If additional lesions were found at CEM compared to the initial routine clinical imaging with digital mammography and ultrasound, a second look ultrasound was immediately performed by the radiologist on a Toshiba Aplio 400 ultrasound system and relevant biopsies were taken. Three projections at CEM were used to correctly identify lesions for ultrasound-guided biopsy.

All lesions at DM and US were given a probability of malignancy code of 1–5 (1 = no abnormalities noted, 2 = benign findings, 3 = non-specific findings with low probability of cancer, 4 = findings suspicious of cancer and 5 = findings highly suspicious of cancer).

This classification cannot be used for CEM images, where only enhancement is given. The different patterns of enhancement can imply a pathologic lesion or benign background enhancement. Extra biopsies were taken from enhanced areas on CEM, which could not be correlated to malignant lesions diagnosed from initial DM and US. No CEM finding was undetectable at the second look ultrasound in this study.

Total extent of the tumour area in millimetres was preoperatively estimated according to a protocol for each imaging modality (DM, US and CEM). The reported extent for each modality expresses the largest measured size across the mass. In case of multifocality, the total extent measures the sum of the largest size of each lesion and the distance between the lesions. This extent and the location of the tumour area is what the surgeon has to take into consideration in planning and performing the surgery. The largest extent of the area on histopathology slides was measured in an identical way and considered as reference standard. Initial DM and US were performed on the same day as part of routine assessment. The CEM was performed median 14 (range 7–31) days after the DM and US.

### Endpoints

The primary endpoint was change in treatment due to CEM findings, defined as mastectomy instead of partial mastectomy (PME) or vice versa, bilateral surgery instead of unilateral due to detection of contralateral cancer and neoadjuvant treatment instead of primary surgery. Secondary endpoints were to perform subgroup analyses of the performance of CEM by age, breast density, cancer subtype and presence of microcalcifications, to see if these factors affect the rate of change in the primary treatment plan. Accuracy of extent estimation of the malignant lesions by different methods (CEM, US and DM) compared to definitive histopathology, and the discrepancy between the methods were assessed. The number of extra biopsies taken due to new findings and quantity of these with malignant results was recorded as well as the number of adverse events to provide a risk evaluation of CEM, especially regarding the injection of iodinated contrast agent.

### Statistical analyses

Descriptive statistics were used for parametric variables expressed as mean/median, standard deviation (SD)/interquartile range (IQR) and range, depending on the distribution. Descriptive statistics for non-parametric variables were presented as frequencies and percentages. Fisher’s exact test was used to assess differences between groups and subgroups when applicable.

Pearson’s correlation coefficient and Bland-Altman statistics for each modality were performed for preoperative size estimation of the malignant changes by imaging modality in comparison with histopathology. Mean and median values of total extent are presented.

Statistical analyses were performed using SAS version 9.4, SAS Institute Inc., Cary, NC, USA.

## Results

The study cohort included 47 women. Median age was 64 years (range 34–82 years), and body mass index (BMI) was 24.8 kg/m^2^ (range 18.1–35.8 kg/m^2^). In 24/47 patients (51%), the malignancy was discovered by mammography screening. Breast density was assessed as low (A or B) in 31/47 patients (66%) and as high (C or D) in 16/47 (34%), according to the BI-RADS® classification (21). All patients in this study had malignancy/suspected malignancy only on one side at inclusion. In 8/47 cases (17%), there was a confirmed multifocality at inclusion (found by DM or US). In 16/47 cases (34%), there was presence of microcalcifications. Prior to inclusion, core-needle biopsies had shown DCIS in 7/47 cases (15%) and invasive cancer in 39/47 (83%). For one patient, there was a suspicion of malignant diagnosis (DM and US malignancy code 5) without positive biopsy. In the study cohort, after standard evaluation with DM and US and before CEM, there was a recommendation of PME in 39/47 patients and mastectomy in 8/47 patients.

**Fig. 1:**
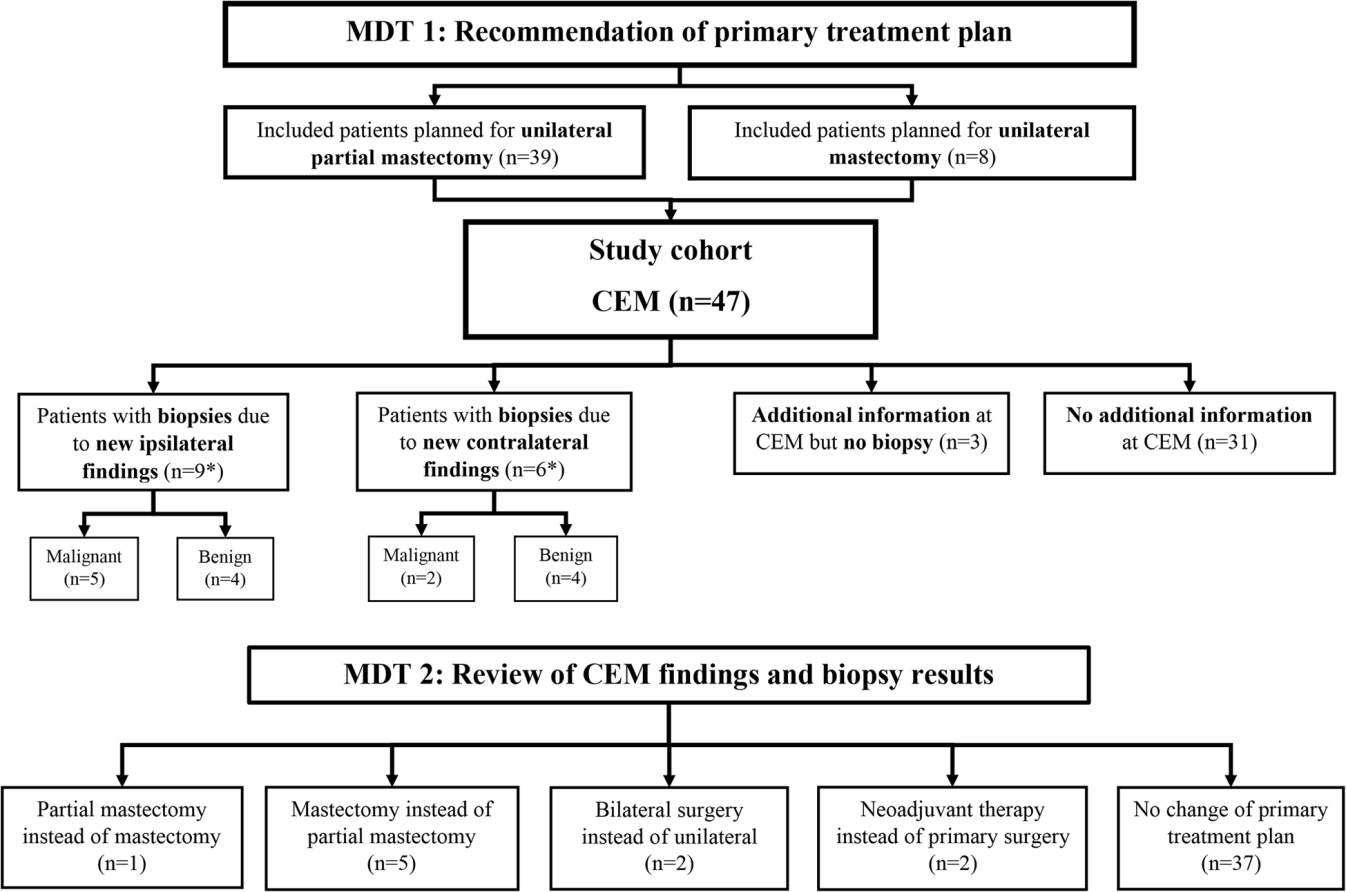
Flowchart of how the treatment plan was affected by CEM-related findings and biopsies. *Two patients had biopsies towards both ipsilateral and contralateral breast after CEM. CEM, contrast-enhanced mammography; MDT, multidisciplinary team conference

The primary treatment changed in 10/47 cases (21%) after CEM. A flowchart of how the treatment was affected by CEM-related findings and biopsies is shown in Fig. 1. For five patients, mastectomy instead of PME was performed, due to finding of multifocal cancer in three patients and due to larger unifocal extent in two patients. For one patient, PME instead of mastectomy was performed, due to improved demarcation of the tumour area. For two patients, bilateral surgery was performed instead of unilateral surgery, due to finding of contralateral cancer. Two patients went to neoadjuvant treatment instead of primary surgery, leaving 45 patients going through primary surgery in the cohort. Four patients were subjected to mastectomy instead of a treatment plan of PME at MDT, in one patient due to her own choice and in three patients due to medical reasons. First operation was subsequently PME in 31/45 (69%) patients (one bilateral) and mastectomy in 14/45 (31%) patients (one bilateral). Four patients (9%) went through a reoperation due to inadequate margins. None of these were among those who had a change in treatment plan after CEM. The result after final surgery was PME in 30/45 patients (67%) (bilateral in one patient) and mastectomy in 15/45 patients (33%) (bilateral mastectomy in one patient).

No differences were seen for the frequency of change in therapy after CEM when the cohort was subgrouped by age, breast density, cancer subtype or presence of microcalcifications (Table 2).

**Table 2:** Subgroup analysis: impact of breast density, age, cancer type and microcalcifications regarding therapy modification after CEM regarding therapy modification

Grouping	*Subgroup*	Therapy modified after CEM		*p* value^2^
		Yes, *n* (%)	No, *n* (%)	
All patients		10 (21)	37 (79)	
Breast density group^1^	*A or B*	8 (26)	23 (74)	
	*C or D*	2 (12)	14 (88)	0.457
Age group^3^	*Below 56*	4 (27)	11 (73)	
	*56 and above*	6 (19)	26 (81)	0.704
Cancer type (index)	*Invasive ductal cancer*	5 (19)	21 (81)	
	*Invasive lobular cancer*	2 (25)	6 (75)	
	*Other invasive cancer*	0	3 (100)	
	*DCIS*	1 (13)	7 (88)	1.0000
Microcalcifications	*No*	5 (17)	24 (83)	
	*Yes*	4 (25)	12 (75)	0.6998

*CEM* contrast-enhanced mammography, *DCIS* ductal carcinoma in situ

^1^Breast density graded according to BI-RADS®, ACR 5th Edition; A–B, low density; C–D, high density

^2^Fisher’s exact test for association between grouping variable and therapy modification

^3^Age of 56 defines an expected cut-off between pre- and postmenopausality

**Fig. 2:**
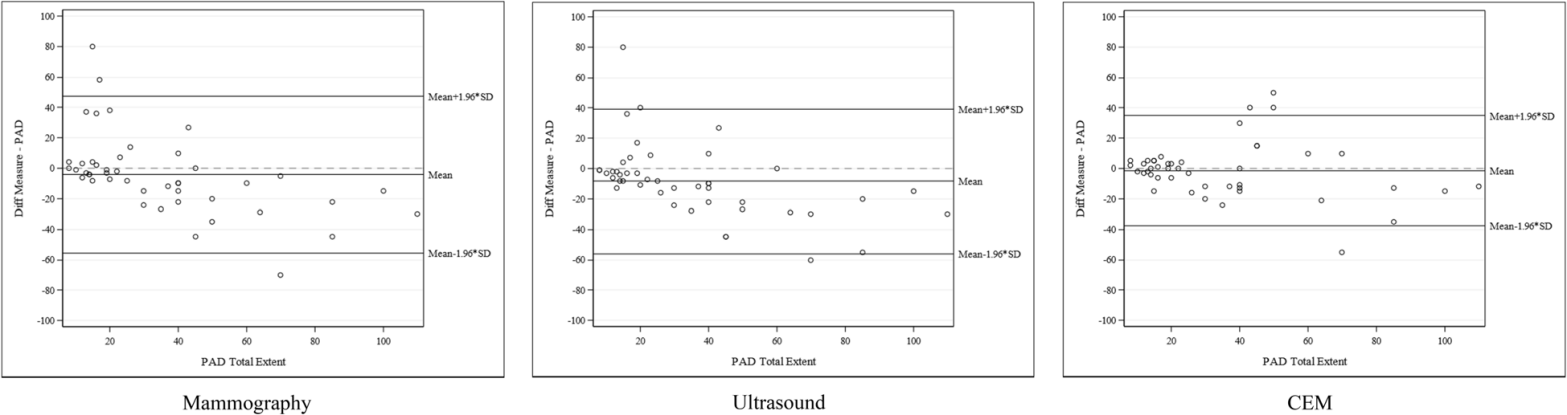
Bland-Altman plots: estimated extent by mammography, ultrasound and CEM compared to histopathology (PAD). Mammography, US and CEM images were compared to histopathological extent (used as the reference value. Mean difference for mammography measurements, − 4.18 mm (95% LOA − 55.534 to 47.179 mm); US, − 8.14 mm (95% LOA − 55.977 to 39.266 mm); and CEM, − 1.36 mm (95% LOA − 37.52 to; 34.812 mm). CEM, contrast-enhanced mammography; LOA, limits of agreement; PAD, pathological anatomical diagnosis

Mean histological extent was 35.1 mm (SD 25.4). Agreement with histopathology was better for CEM (Bland-Altman statistics; mean difference − 1.36, SD ± 18.45) regarding preoperative size estimation of the malignant changes in comparison with mammography (− 4.18, SD ± 26.20) and ultrasound (− 8.36, SD ± 24.30) (Table 3, Fig. 2). Pearson’s correlation coefficients between the extent estimated by CEM and the definitive histopathological extent were between 0.769 and 0.915 in subgroup analyses of type of cancer (invasive ductal/invasive lobular/DCIS), and with and without microcalcifications, compared to extent estimates for DM of 0.151 and 0.647 and US of 0.389 and 0.670 (Table 4).

**Table 3:** Estimation of total extent for all modalities and compared to histopathology

Method		Total extent (mm)	Difference from histopathology^1^ (mm)	LOA^2^ (mm)
CEM	*Mean (SD)*	33.8 (28.3)	− 1.4 (18.5)	− 37.523; 34.812
	*Median (Q1; Q3)*	22 (14; 50)	0 (− 12; 5)	
	*Min; max*	0^3^; 100	− 55; 50	
Ultrasound	*Mean (SD)*	26.8 (24.2)	− 8.4 (24.3)	− 55.977; 39.266
	*Median (Q1; Q3)*	18 (9; 35)	− 8 (− 22; − 1)	
	*Min; max*	0^3^; 95	− 60; 80	
Mammography	*Mean (SD)*	31.0 (24.8)	− 4.2 (26.2)	− 55.534; 47.179
	*Median (Q1; Q3)*	20 (12; 50)	− 5 (− 15; 3)	
	*Min; max*	0^3^; 95	− 70; 80	
Histopathology	*Mean (SD)*	35.1 (25.4)		
	*Median (Q1; Q3)*	26 (15; 45)		
	*Min; max*	8; 110		

Difference is calculated as follows: Total extent from estimation method − total extent from histopathology

*CEM* contrast-enhanced mammography

^1^Bland-Altman statistics (for plots, see Fig. 2)

^2^Limits of agreement: mean diff ± 1.96 × SD

^3^Value of 0 was given for lesions that were not detected by the imaging modality

**Table 4:** Correlation of estimated extent in relation to histopathological extent

Subgroup		*n*	Mammography	Ultrasound	CEM
Cancer type	*Invasive ductal cancer*	26	0.533	0.648	0.818
	*Invasive lobular cancer*	8	0.400	0.453	0.835
	*DCIS*	8	0.151	0.389	0.769
Microcalcifications	*No*	28	0.647	0.670	0.770
	*Yes*	15	0.366	0.474	0.915

Pearson’s correlation coefficient was used to compare extent for all modalities (CEM, ultrasound and mammography) to definitive extent from the postoperative histopathological report. Pearson’s correlation coefficient measures the strength of the monotonic relationship between continuous data and may lie between − 1 and 1. 0–0.19 = very weak, 0.2–0.39 = weak, 0.4–0.59 = moderate, 0.6–0.79 = strong, 0.8–1 = very strong

*CEM* contrast-enhanced mammography, *DCIS* ductal carcinoma in situ

In total, 19 additional lesions in 13/47 patients (28%) were biopsied due to detection at CEM (Table 5). In nine of the thirteen patients, only one lesion was biopsied, but in four of them, two or more lesions were biopsied. Nine of the 19 biopsied lesions showed malignant disease (6 invasive cancer (32%) and 3 DCIS (16%)). Ten biopsied lesions were subsequently benign.

**Table 5:** Additional biopsies due to findings from CEM

At least one additional biopsy (*n* = 47)	*No*	34 (72%)
	*Yes*	13 (28%)
Biopsy led to changed treatment (*n* = 13)	*No*	6 (46%)
	*Yes*	7 (54%)
Additional biopsies per patient	*0*	34 (72%)
	*1*	9 (19%)
	*2*	2 (4%)
	*3*	2 (4%)
Total number of additional biopsies	19	
Outcome from additional biopsies (*n* = 19)	*Invasive cancer*	6 (32%)
	*DCIS*	3 (16%)
	*Benign*	10 (53%)

*CEM* contrast-enhanced mammography, *DCIS* ductal carcinoma in situ

There were no adverse events during the CEM procedure. In three patients (6%), dizziness, light nausea and warmth (symptoms well recognized after injection of iodinated contrast agent) were recorded but went spontaneously in total regress within a few minutes.

## Discussion

The results from this feasibility study indicate that CEM has an added value in the preoperative setting. For 21% of the evaluable patients, the primary treatment was changed due to CEM findings. Importantly, no major adverse events occurred and only minor inconveniences after injection of iodinated contrast agent were recorded, which holds promise for future studies.

Our result is in concordance with two retrospective reviews of the impact of CEM. In a study by Tardivel et al., CEM changed diagnostic and treatment strategy in 41/195 (21%) of cases with suspicious and undetermined findings on DM and/or US in post-screening assessment, either by more extensive surgery or neoadjuvant therapy in cases with additional malignant lesions at CEM or by avoiding further biopsy in cases with negative CEM [10]. Ali-Mucheru et al. made a retrospective review of 101 patients who had surgery for breast malignancy and found that the surgical procedure had changed in 20 cases (20%) after additional CEM [19]. In that cohort, 14 patients had neoadjuvant therapy and 41 patients also had an MRI performed.

No differences in the proportion of change in treatment were seen in our study, when patients were subgrouped by age, breast density, cancer subtype or presence of microcalcifications. This indicates that CEM has added value for a large spectrum of patients with malignant breast lesions. As this study only includes a small number of individuals, a larger study is needed to assess these endpoints. However, results from this feasibility study stipulate no indication to limit the application of CEM to a certain subgroup, in the future trial.

In this study, CEM was superior to both US and DM regarding extent estimation, as random measurement errors for CEM were smaller than those for DM or US. All modalities tended to underestimate the extent compared to histological extent; however, the mean difference for CEM was closest to histopathology even in the presence of microcalcifications. A reservation has to be made in this aspect as for larger tumours, US may have challenges to correctly measure the tumour extent if it goes beyond the transducer width of 5 cm.

CEM is presented as an alternative to MRI. In both methods, contrast medium uptake is pronounced in malignant lesions. Sensitivity of MRI is excellent; however, a varying specificity has been reported for this modality (47–97%) [22]. Previous studies of CEM have indicated a sensitivity similar to that of MRI [23–25], and equivalent or even higher specificity as well as equivalent tumour extent measurement [14]. CEM was not compared to MRI in our study. However, similar percentage for therapy modification as that found in this study was presented in a study assessing the added value of MRI, where the primary treatment plan was changed for 18% of the study population [26].

A negative aspect of CEM is that it yields an additional dose of radiation to the women; previous studies have reported an increase in average glandular dose (AGD) of 42–80%, compared to DM [27]. However, AGD from CEM is below the maximum dose regulated in the Mammography Quality Standards Act [13, 28, 29]. In this study, three projections of each breast were calculated to give each woman an extra radiation dose of maximum 0.75 mSv in total.

MRI does not yield additional radiation; however, there is unclearness regarding eventual long-term effects of the contrast agent gadolinium, which has been found to accumulate in the brain (dentate nuceli, globus pallidus) [30]. Both iodinated contrast agent and gadolinium have side effects to the renal system with potential nephrotoxicity [31].

MRI is a resource-demanding method. Availability may vary for the modality itself, and MRI-guided biopsy is not accessible in many units. Due to the position of the patient during MRI, findings can be difficult to identify for US-guided biopsy afterwards. CEM is performed within a few minutes on a mammography apparatus using standard DM projections. Findings are thereby easy to identify by US for guidance of a biopsy, especially if both CC and ML projections are included in the protocol.

Strengths of this study include that two radiologists evaluated all imaging modalities (DM, US and CEM). There was also a good representation of ages, breast density and tumour types in the cohort of study patients. As patients were consecutively included, we believe that selection bias is low in this cohort.

A limitation of the study is the small cohort size, although large enough in the preparation for a future trial. Furthermore, DM and US were performed in the regular clinical setting and more defined protocols are needed for size estimation from DM and US to limit impact of individual radiologists (especially for US as DM can be reviewed retrospectively). Since this is a feasibility study, it was not possible to have a blinded size assessment for the different modalities.

The included patients were the first to undergo CEM in the present hospital. This may reflect the rate of benign biopsies after findings with CEM in this study. Ten of the 19 biopsies were benign and may be considered as false-positive findings of CEM. Benign biopsies can however be helpful in the preoperative evaluation to limit malignant extent, allowing treatment recommendation to change from mastectomy to PME. Additionally, some benign biopsies occurred due to finding of diffuse contrast enhancement, which has been previously studied in contrast-enhanced MRI [32]. This is often found in premenopausal women and can be hard to distinguish from DCIS. It can often be avoided by timing the imaging diagnostics in relation to the patient’s menstrual cycle [32]. However, in the preoperative staging of breast cancer, there is no time to await ideal timing. Unnecessary biopsies are however of nuisance to the patient and should be avoided if possible. Plausibly, the identification of diffuse contrast enhancement compared to malignant findings will improve, as the radiologists become more acquainted with reading of CEM.

By running this feasibility study, routines were set up and experiences gained, providing a good setting for the future prospective randomized trial. The future trial will assess all endpoints included in this study and more thoroughly explore the potential impact of additional preoperative CEM regarding number of reoperations, possible avoidance of mastectomy, margin status of PMEs, 5-year recurrence rates and patient-reported health-related quality of life. In addition, a cost/benefit evaluation of CEM will be performed.

## Conclusion

In this feasibility study, CEM has shown an added value in preoperative staging of malignant breast lesions regarding impact on primary treatment by improved demarcation and extent estimation of tumours, finding of contralateral cancer and multifocality. This feasibility study is a foundation for a planned prospective randomized trial, exploring the added value of CEM in preoperative staging of breast cancer patients. Importantly, no major adverse events were seen, and only three patients experienced slight inconveniences after injection of the contrast medium.

## Data Availability

The datasets generated and/or analysed during the current study are available from the corresponding author upon reasonable request.

## Abbreviations

AGD: Average glandular dose
BMI: Body mass index
CC: Cranio-caudal
CEM: Contrast-enhanced mammography
DCIS: Ductal carcinoma in situ
DM: Digital mammography
ER: Estrogen receptor
IQR: Interquartile range
LOA: Limits of agreement
MDT: Multidisciplinary team conference
ML: Mediolateral
MLO: Mediolateral-oblique
MRI: Magnetic resonance imaging
PgR: Progesterone receptor
PME: Partial mastectomy
SD: Standard deviation
TSH: Thyroid stimulating hormone
US: Ultrasound
